# The Role of the SOS Response in the Adaptation of *Pseudomonas aeruginosa*

**DOI:** 10.3390/cimb48040355

**Published:** 2026-03-28

**Authors:** Emilia Zarembska, Anna Pietruczuk-Padzik, Małgorzata Wrzosek

**Affiliations:** 1 Student Scientific Association “FARMAKON”, Medical University of Warsaw, 1 Banacha St., 02-097 Warsaw, Poland; 082906@student.wum.edu.pl; 2 Department of Pharmaceutical Microbiology and Bioanalysis, Medical University of Warsaw, 02-097 Warsaw, Poland; anna.pietruczuk-padzik@wum.edu.pl; 3 Department of Pharmaceutical Sciences, Medical College, Jan Kochanowski University, 25-369 Kielce, Poland; 4 Department of Biochemistry and Pharmacogenomics, Medical University of Warsaw, 02-097 Warsaw, Poland

**Keywords:** *Pseudomonas aeruginosa*, SOS response, RecA–LexA regulatory system, antibiotic-induced mutagenesis, fluoroquinolones, antibiotic resistance

## Abstract

*Pseudomonas aeruginosa* is a major opportunistic pathogen whose adaptive capacity limits the long-term efficacy of antibiotic therapy. Beyond classical resistance mechanisms, antibiotics may also act as stress signals that alter bacterial physiology and evolutionary trajectories. A central element of this response is the SOS regulatory network, controlled by the RecA–LexA system. Although well studied in *Escherichia coli*, SOS signaling in *P. aeruginosa* shows distinct regulatory features that remain incompletely understood. This review summarizes experimental and clinical evidence on antibiotic-induced SOS responses in *P. aeruginosa*, focusing on fluoroquinolones and other genotoxic agents. Fluoroquinolone exposure consistently induces SOS activation and RecA-dependent signaling, affecting short-term antibiotic susceptibility. However, the available evidence does not support a universal role for SOS activation as a major driver of long-term resistance evolution under most tested conditions. Its relationship with antibiotic-induced mutagenesis remains variable: some studies implicate low-fidelity DNA polymerases, whereas others report mutagenesis independent of canonical RecA–LexA control. Beyond mutagenesis, SOS activation may affect integron dynamics, virulence, and biofilm-associated phenotypes. Overall, in *P. aeruginosa*, the SOS response appears to be a context-dependent modulator of stress adaptation rather than a universal determinant of resistance evolution.

## 1. Introduction

The rapid emergence of antibiotic resistance among bacterial pathogens represents one of the most pressing challenges in contemporary medicine. Among Gram-negative bacteria, *Pseudomonas aeruginosa* occupies a particularly critical position due to its intrinsic resistance to multiple classes of antimicrobials, remarkable metabolic plasticity, and exceptional ability to adapt to the hostile environments encountered during infection and antimicrobial therapy. This opportunistic pathogen is a leading cause of hospital-acquired infections, including ventilator-associated pneumonia, and is particularly important in patients with cystic fibrosis. Indeed, approximately 60–70% of adults with cystic fibrosis are chronically colonized by *P. aeruginosa* [1]. This bacterium is also a common cause of bloodstream and chronic wound infections, particularly in immunocompromised individuals and patients with metabolic disorders, including diabetes mellitus. Infectious foot ulcers are the most common, severe, and economically burdensome complication of type 2 diabetes, with approximately 60% of diabetic foot ulcers becoming infected [2]. Recurrent infections, particularly those associated with chronic, non-healing wounds requiring repeated hospitalizations, are linked to an increased prevalence of treatment-resistant Gram-negative bacteria [3].

The growing incidence of multidrug-resistant (MDR) *Pseudomonas aeruginosa* strains severely limits available therapeutic options and highlights the urgent need to identify alternative and adjunctive strategies to support the rapidly diminishing antimicrobial arsenal. One of the key drivers of antibiotic resistance evolution is the widespread presence of antimicrobial compounds in the environment at subinhibitory concentrations that do not inhibit bacterial growth. Such contamination not only exerts selective pressure but also profoundly affects bacterial gene expression, promotes biofilm formation, and enhances mutagenesis, ultimately contributing to the development of both genotypic and phenotypic resistance [4,5]. Bacterial stress response mechanisms play a pivotal role in adaptation to antibiotic pressure; therefore, understanding these responses is essential for developing novel therapeutic approaches. Antibiotics have traditionally been regarded as agents that inhibit essential bacterial processes such as cell wall synthesis, protein translation, or DNA replication. However, accumulating evidence demonstrates that antibiotics also act as potent modulators of bacterial physiology, activating complex stress response networks that extend far beyond their primary molecular targets. Sublethal or transient exposure to antibiotics can induce extensive changes in gene expression, metabolic pathways, and cellular architecture, thereby reshaping bacterial phenotypes and evolutionary trajectories. In this context, antibiotics not only select for pre-existing resistant variants but may also actively promote the generation of genetic and phenotypic diversity that fuels bacterial adaptation. Among bacterial stress response systems, the SOS regulon represents a particularly critical and well-characterized pathway. The available literature strongly links SOS activation to mutagenesis and antibiotic resistance development [6]. Nevertheless, substantial interspecies differences exist in both the physiology and functional significance of this system. The SOS response was originally described in *Escherichia coli*, where its activation was associated primarily with genotoxic stress and the accumulation of DNA damage. In other bacterial species, including *P. aeruginosa*, the regulation, functional roles, and clinical implications of SOS activation appear to differ considerably. Therefore, this review aims to compile and critically summarize the current knowledge on the relationship between antibiotic therapy and molecular stress responses in *P. aeruginosa*. Particular attention is given to the molecular architecture of the SOS regulon, the role of error-prone DNA polymerases in mutagenesis, and the consequences of SOS activation for mutation rates, antibiotic resistance development, virulence, and biofilm formation. A comprehensive understanding of these molecular mechanisms and the metabolic pathways affected by antibiotic exposure is essential for the rational design of therapies that directly target stress response components and mutagenic processes. Such targeted strategies may ultimately help to curb the spread of antibiotic resistance and attenuate bacterial virulence. Collectively, the available data indicate that the SOS response in *P. aeruginosa* differs fundamentally from the canonical *E. coli* paradigm. Rather than acting as a universal mutagenic engine, SOS activation appears to function as a context-dependent regulator of stress adaptation, phenotypic diversification, and virulence.

**Figure 1:**
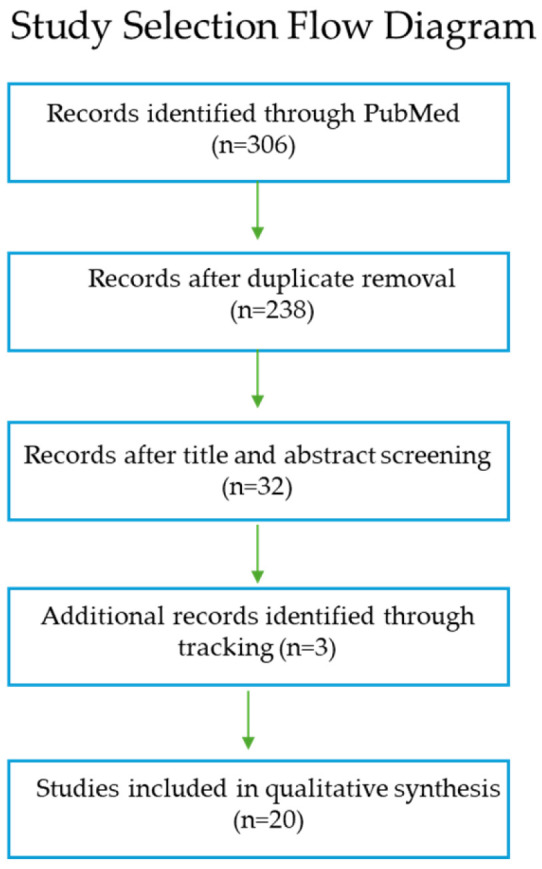
Flow diagram of the literature search and study selection process. Records were identified through a PubMed database search, followed by the duplicate removal and screening of titles and abstracts. Additional studies were retrieved through citation tracking. Studies meeting the inclusion criteria were subsequently included in the qualitative synthesis.

A literature search was conducted in the PubMed/MEDLINE database to identify studies investigating the antibiotic-induced SOS response in *P. aeruginosa*. Two complementary search strings were applied independently: “*Pseudomonas aeruginosa*” AND (SOS OR RecA OR LexA OR “DNA damage response”), and “*Pseudomonas aeruginosa*” AND (RecA OR LexA OR SOS) AND (antibiotic OR fluoroquinolone OR ciprofloxacin). These strategies were selected to capture both general SOS pathway activation and antibiotic-specific induction. All the retrieved records were imported into a single reference management library, and duplicate entries were identified and removed [7]. A prescreening step was subsequently applied to refine the dataset. Titles and abstracts were evaluated for relevance to *P. aeruginosa* and the SOS response. Records clearly unrelated to SOS regulation, DNA damage signaling, or antibiotic exposure were excluded at this stage. To ensure the comprehensive coverage of seminal and highly relevant literature, additional studies not retrieved through the database search were identified through the manual citation tracking of key publications. These records were added to the dataset prior to full-text evaluation and were treated equivalently to database-derived studies. The full-text versions of all prescreened articles were independently assessed for eligibility. Studies were included if they met the following criteria: primary experimental research, investigation of the SOS response or its core components (RecA, LexA, or SOS-regulated genes), use of *P. aeruginosa* as the model organism, and assessment of antibiotic-induced or DNA damage-associated SOS activation. Review articles, conference abstracts, and studies lacking primary experimental data were excluded. Due to heterogeneity in experimental designs, inducing agents, and outcome measures, a quantitative meta-analysis was not performed. Instead, the findings were synthesized qualitatively with emphasis on the molecular mechanisms of SOS activation, antibiotic-specific effects, and biological consequences relevant to adaptation, mutagenesis, and antimicrobial tolerance. The selection process is summarized in a PRISMA-compliant flow diagram [7], (Figure 1).

## 2. Molecular Basis of the SOS System

**Figure 2:**
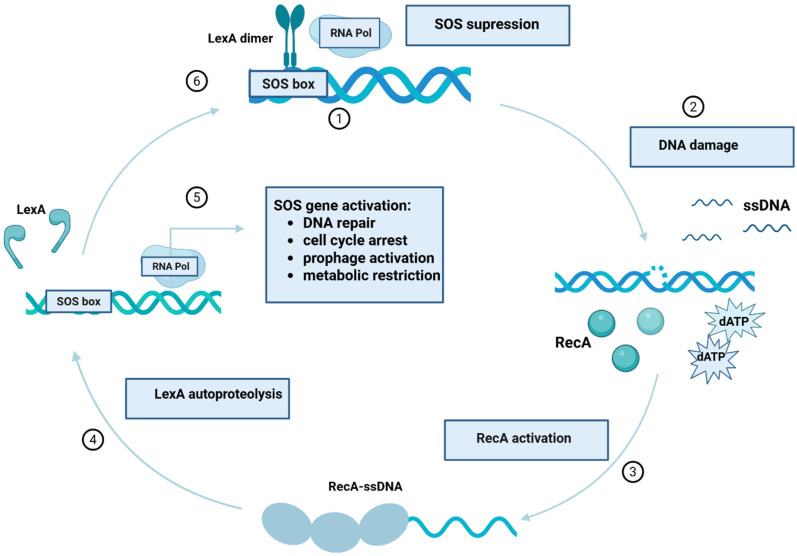
DNA damage-induced activation of the SOS response. 1. In the absence of cellular stressors, *lexA* expression is maintained at a basal level, ensuring the continuous repression of SOS regulon genes; 2. DNA damage leads to replication fork arrest and results in the accumulation of single-stranded DNA (ssDNA); 3. RecA binds to ssDNA and, in the presence of (d)ATP, becomes activated; 4. the activated RecA protein interacts with LexA, promoting its autocatalytic cleavage; 5. relief from LexA-mediated repression allows for the transcription of SOS system genes; and 6. following DNA repair, LexA reassociates with operator sequences located near the promoters of SOS regulon genes, leading to the attenuation of the SOS response. Based on [6,12]. ssDNA—single-stranded DNA; (d)ATP—deoxyadenosine triphosphate. Created in BioRender. Biochemii, K. (2026) https://BioRender.com/ep9eas1 (accessed on 25 February 2026).

The SOS response represents one of the most evolutionarily conserved global regulatory networks in bacteria, dedicated to sensing and mitigating genotoxic stress. First described in 1975 by Radman in *Escherichia coli*, the SOS system was initially proposed as an inducible DNA repair pathway that enables bacterial survival following extensive genomic damage, while simultaneously promoting mutagenesis [8]. The emergence of such a system reflects the constant exposure of bacterial genomes to endogenous threats, including replication errors and reactive oxygen species, as well as exogenous insults such as ultraviolet radiation (UV), chemical mutagens, and antimicrobial agents. At the core of the SOS response lie two proteins with antagonistic yet tightly coordinated functions: the recombinase RecA and the transcriptional repressor LexA. Under physiological, no-stress conditions, LexA dimers bind to conserved operator sequences, commonly referred to as SOS boxes, located in the promoter regions of SOS-regulated genes, thereby repressing their transcription [9]. The sequence and architecture of these operator sites are conserved within phylogenetic groups but differ between distant bacterial lineages, underscoring the evolutionary divergence of SOS regulatory logic [10]. The identified consensus sequence for LexA binding in *P. aeruginosa* (CTG-TATAA-ATATA-CAG) is closely similar to that described in *E. coli*, except position 8, where *E. coli* more frequently possesses a deoxythymidine-5′-monophosphate (dTMP) [11]. Genotoxic stress that interferes with DNA replication leads to replication fork stalling and the accumulation of single-stranded DNA (ssDNA). RecA rapidly binds ssDNA in an ATP-dependent manner, forming nucleoprotein filaments that acquire co-protease activity. These activated RecA filaments stimulate the autocatalytic cleavage of LexA, leading to the derepression of the SOS regulon [9]. Consequently, a temporally orchestrated transcriptional program is initiated, encompassing genes involved in high-fidelity DNA repair, cell cycle control, recombination, prophage induction, and, ultimately, error-prone DNA synthesis [12]. Figure 2 illustrates the molecular mechanism of the SOS response. A defining feature of the SOS response is its hierarchical activation. In *E. coli,* early stages are dominated by high-fidelity repair mechanisms, such as nucleotide excision repair (NER) and homologous recombination (HR), as well as transient inhibition of cell division mediated by SulA-dependent blockade of FtsZ polymerization [9]. This growth arrest provides the cell with time to repair damaged DNA before resuming replication. If damage persists or becomes extensive, late SOS genes encoding translesion DNA synthesis (TLS) polymerases are induced. These low-fidelity polymerases enable replication across damaged templates but do so at the cost of increased mutation rates, thereby introducing genetic variability [13].

### 2.1. Different Faces of the SOS Response

The SOS system is widely distributed across the bacterial domain and is considered the most conserved DNA damage response known in prokaryotes. Homologs of *lexA* have been identified in nearly all sequenced bacterial genomes, with notable exceptions such as *Bacteroidetes*, *Chlorobi*, and *Epsilonproteobacteria*. In certain species, including *Streptococcus pneumoniae*, the classical SOS system is absent and replaced by alternative damage response networks centered on DNA uptake and recombination [10]. These variations illustrate that although the need to respond to DNA damage is universal, the molecular strategies employed are shaped by the ecological niche, genomic architecture, and evolutionary history. Importantly, although the general framework of the SOS response is conserved, its composition, regulatory scope, and physiological consequences vary substantially between species. In *E. coli*, approximately 43 genes are under LexA control. In contrast, the SOS regulon of *P. aeruginosa* comprises only 15 genes, highlighting a distinct regulatory architecture compared to canonical enterobacterial models [11]. Despite this variability, three components are universally conserved across all SOS systems: the RecA activator, the LexA repressor, and at least one error-prone DNA polymerase [14]. This conservation underscores mutagenesis as a fundamental evolutionary function of the SOS response. The widespread distribution of translesion synthesis (TLS) polymerases across diverse bacterial species underscores the critical role of this process in the genotoxic stress response. By enabling short-range replication across damaged DNA lesions in the absence of a correct template, TLS allows for the temporary continuation of cellular growth until more sophisticated genome repair pathways are recruited. This adaptive strategy, however, comes at the cost of elevated replication error rates, resulting in an increased mutational burden.

In *E. coli*, the induction of mutagenesis is mediated primarily by DNA polymerase V (Pol V), encoded by the *umuDC* operon, whose activation occurs approximately 20 min after SOS initiation [15]. Pol V assembly requires RecA-dependent proteolytic processing of UmuD to UmuD′, followed by association with UmuC. In parallel, DNA polymerase IV (DinB) and polymerase II (PolB) are also upregulated, collectively contributing to lesion bypass and increased mutation frequency [16]. *P. aeruginosa*, however, deviates substantially from this paradigm. The genome lacks homologs of the *umuDC* operon, and the regulation of TLS polymerases differs fundamentally from that observed in enterobacteria [11]. Studies employing *P. aeruginosa* as a model organism have produced heterogeneous and sometimes conflicting results. These discrepancies may depend on the nature of the stress-inducing agent, the duration of the stress response, and the specific characteristics of the strain or isolate under investigation. Consequently, the outcome of alarm response activation may be heterogeneous and therefore difficult to define unequivocally. These findings suggest that the classical view of SOS-driven mutagenesis, derived largely from *E. coli*, represents only one manifestation of a broader and more diverse regulatory landscape. In *P. aeruginosa*, SOS activation may play a more nuanced role, allowing for a balance between survival, mutagenesis, and virulence. Beyond mutagenesis, the SOS response exerts pleiotropic effects on bacterial physiology. It influences cell cycle progression, metabolic reprogramming, biofilm maturation, virulence factor expression, and prophage induction [12]. The induction of prophages and the mobilization of genetic elements during SOS activation establish a direct link between DNA damage responses and horizontal gene transfer (HGT), facilitating the dissemination of virulence and resistance determinants across bacterial populations. Thus, the SOS response should not be viewed as a monolithic pathway but rather as a modular and context-dependent regulatory network whose functional output varies according to the species, environmental conditions, and nature of the inducing stress.

### 2.2. Antibiotics as Modulators of Bacterial Genotype and Phenotype

Antibiotics have traditionally been classified based on their primary molecular targets, such as cell wall biosynthesis, protein translation, or DNA replication. However, it is now evident that antimicrobial agents exert far broader effects on bacterial cells, functioning as potent signaling molecules that reshape global gene expression, metabolism, and cellular behavior. These effects draw special attention to subinhibitory concentrations, which are widespread in clinical settings, host tissues, and the environment.

Transcriptomic studies have demonstrated that exposure to antibiotic concentrations well below the MIC induces the extensive reprogramming of bacterial gene expression. In *P. aeruginosa*, sublethal ciprofloxacin exposure alters the expression of hundreds of genes, including those involved in DNA replication, cell division, membrane permeability, motility, and energy metabolism. Notably, such exposure leads to the downregulation of energetically costly processes while selectively modulating virulence-associated pathways. One striking example is the dramatic upregulation of *ptrB*, encoding a repressor of the type III secretion system (T3SS) [11]. This response reflects a strategic shift toward energy conservation under stress conditions, with the reduced production of virulence factors and prioritization of survival. Concurrently, antibiotic exposure induces the strong expression of prophage-associated genes, reinforcing the link between antibiotic stress, SOS activation, and HGT.

Importantly, antibiotics that do not directly target DNA can also induce genotoxic stress indirectly. Aminoglycosides, β-lactams, and tetracyclines promote the accumulation of reactive oxygen species, leading to oxidative DNA damage and activation of stress response pathways, including the SOS system. Moreover, antibiotics can perturb nucleotide pools, translational fidelity, and redox homeostasis, further contributing to genomic instability [4]. These observations challenge the simplistic dichotomy of bactericidal versus bacteriostatic drugs and underscore the role of antibiotics as global modulators of bacterial genotype and phenotype. By altering mutation rates, virulence expression, biofilm formation, and stress tolerance, antibiotics may both select for pre-existing resistant clones and actively shape bacterial evolutionary trajectories.

### 2.3. SOS System and Antibiotic Resistance

Antibiotic resistance is a direct consequence of bacterial evolutionary plasticity, driven by processes like mutation, selection, and horizontal gene transfer [17]. The SOS response occupies an important position at the intersection of these processes, linking DNA damage sensing with mutagenesis and diversification. In model organisms such as *E. coli*, the role of the SOS system in resistance evolution is well established. SOS induction enhances mutation rates, facilitates recombination-dependent DNA repair, and promotes the emergence of resistance-conferring mutations following fluoroquinolone exposure [18]. Studies using the *E. coli* model indicate that mutations contributing to ciprofloxacin resistance arise not merely from stochastic replication errors but from the activation of tightly regulated bacterial systems. The activation of these pathways induces the expression of genes encoding protein products that directly participate in mutagenic processes. Moreover, bacteria harboring mutations in key genes involved in DNA repair display an increased susceptibility to ciprofloxacin [19].

**Figure 3:**
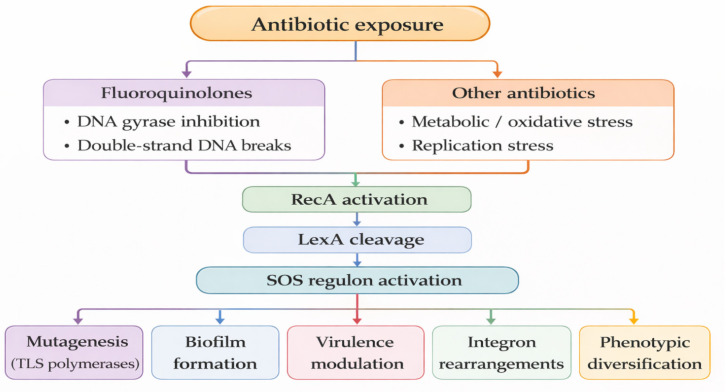
Antibiotic-induced stress pathways leading to SOS activation in *Pseudomonas aeruginosa*.

In *P. aeruginosa*, however, the contribution of the SOS response to antibiotic resistance remains contentious. While fluoroquinolones robustly induce SOS signaling and RecA activity, some studies indicate that resistance-associated mutagenesis can occur independently of LexA, RecA, or canonical TLS polymerases. Mutants lacking RecA exhibit markedly increased susceptibility to fluoroquinolones, underscoring the importance of RecA for survival under genotoxic stress, yet they do not consistently display reduced mutation frequencies [20]. The results of several studies clearly challenge the universal concept derived from *E. coli* model systems, which posits that inhibition of RecA and the SOS response effectively blocks antibiotic-induced mutagenesis. Accumulating evidence suggests that the SOS system may instead contribute indirectly to the emergence of antibiotic resistance by promoting survival under stress conditions, facilitating horizontal gene transfer, and enhancing phenotypic diversification, rather than functioning as a universal mutagenic engine. The available studies linking antibiotic exposure to SOS-dependent mutagenesis in *P. aeruginosa* are analyzed, with particular attention paid to the impact of antibiotic-associated genotoxic stress on virulence, biofilm formation, and the emergence of persister cells. These processes are of substantial clinical relevance, as a comprehensive understanding of the mechanisms underlying antibiotic resistance acquisition and the persistence of chronic infections is essential for the development of effective and rational therapeutic strategies and for reducing the overall burden associated with antimicrobial treatment. Fluoroquinolones represent a particularly relevant class of antibiotics in this context. By stabilizing DNA–topoisomerase complexes, these agents induce double-strand DNA breaks and are among the most potent inducers of the SOS response. There is substantial evidence that even subinhibitory concentrations of fluoroquinolones have been shown to robustly activate SOS signaling, increase mutational frequencies, and modulate diverse phenotypes, including biofilm formation, virulence factor production, and membrane vesiculation [21,22]. Importantly, the SOS response can also be activated indirectly by antibiotics that do not target DNA, such as metronidazole, through redox-mediated genotoxic stress, revealing unexpected routes by which antimicrobial therapy may inadvertently promote resistance development. Collectively, these observations challenge the simplistic view of the SOS response as a uniform driver of antibiotic resistance and highlight its multifaceted role in shaping bacterial adaptation. Understanding how different classes of antibiotics engage the SOS network in *P. aeruginosa*, and how this engagement influences mutagenesis, phenotypic plasticity, and clinical outcomes, is essential for the rational design of therapeutic strategies that not only eradicate bacteria but also limit their evolutionary potential. The current evidence on antibiotic-induced SOS responses in *P. aeruginosa* highlights their complex molecular mechanisms, adaptive consequences, and implications for antimicrobial therapy. Antibiotic exposure can initiate distinct stress pathways that ultimately converge on activation of the SOS regulatory network. In particular, fluoroquinolones induce direct DNA damage through inhibition of DNA gyrase, resulting in the formation of double-strand DNA breaks and the generation of genotoxic stress. In contrast, other classes of antibiotics may activate the SOS response indirectly by inducing metabolic or oxidative stress, replication stress, and secondary DNA damage. The relationships between antibiotic exposure, stress responses, and downstream consequences of SOS activation are summarized in Figure 3. These signals converge on RecA activation and LexA autocleavage, leading to the derepression of the SOS regulon. The subsequent SOS activation influences multiple adaptive processes, including mutagenesis mediated by translesion synthesis polymerases, biofilm formation, virulence modulation, integron rearrangements, and broader phenotypic diversification.

## 3. Induction of the SOS Response and Global Changes in Gene Expression Following Antibiotic Exposure

Fluoroquinolones represent the strongest and most consistent inducers of the SOS response in *P. aeruginosa.* These chemotherapeutic agents exert bactericidal activity by targeting DNA gyrase and topoisomerase IV, leading to the accumulation of enzyme-associated DNA strand breaks through the inhibition of the DNA re-ligation step of the topoisomerase reaction cycle. The resulting DNA damage constitutes a strong intracellular signal that activates RecA and initiates the SOS regulatory cascade. Table 1 summarizes the major published studies investigating the SOS response and antibiotic-induced mutagenesis in *P. aeruginosa*, including experimental models, inducing agents, and proposed molecular mechanisms. Early foundational studies provided direct evidence that the *recA* gene in *P. aeruginosa* is inducible by genotoxic stress, including ultraviolet irradiation and quinolone exposure, thereby establishing the existence of a functional SOS-like regulatory system in this species. The induction of *recA* expression was shown to be time dependent, reaching maximal levels three hours after UV and at a norfloxacin concentration of 1 µg/mL (two-fold higher than MIC) [23]. Notably, this induction was strictly dependent on the presence of a functional RecA protein, consistent with an autoregulatory mechanism characteristic of SOS responses.

Transcriptomic studies performed using ciprofloxacin at clinically relevant concentrations have demonstrated that the SOS response in *P. aeruginosa* comprises a narrow and well-defined regulon that is markedly smaller than that described in *E. coli* [11]. Among the several thousand analyzed genes, only 15 exhibited unequivocal LexA-dependent regulation. These genes were predominantly associated with DNA repair functions, including *recA*, *recN*, and *recX*, as well as with inducible DNA polymerases genes such as *imuB* and *dnaE2*. In contrast, the vast majority of transcriptional changes observed following ciprofloxacin exposure were independent of the SOS response and reflected a global suppression of core cellular processes. Notably, decreased expression was detected for genes involved in cellular respiration (*atpC*, *atpD*), cell division (*ftsZ*, *ftsA*), lipopolysaccharide biosynthesis (*wbp*), DNA replication (*dnaA*, *dnaN*, *gyrB*), and motility (*pilA*). Collectively, these changes are consistent with a broad metabolic and proliferative slowdown rather than a canonical SOS-driven transcriptional program. A particularly striking feature of the ciprofloxacin-induced transcriptome was the pronounced upregulation of the transcriptional regulator PtrR and genes under its direct control. Among these, *ptrB*, which encodes a negative regulator of the type III secretion system (T3SS), exhibited a robust induction, increasing by up to 166-fold. This early finding suggests that ciprofloxacin-triggered DNA damage not only affects genome maintenance pathways but also profoundly reshapes regulatory circuits linked to virulence [11].

The impact of ciprofloxacin on the expression of *dinB*, encoding a translesion synthesis (TLS) DNA polymerase potentially involved in stress-induced mutagenesis, remains less clear. While Cirz et al. reported induction of *dinB* expression in response to ciprofloxacin [11], a subsequent study by Blázquez and coworkers did not observe such induction [24]. Importantly, these discrepancies can be largely attributed to differences in antibiotic dosage, as the dinB induction observed by Cirz et al. was detected at ciprofloxacin concentrations approximately 25 times higher than those used by Blázquez et al. in the latter study. Moreover, transcriptional analyses by Cirz et al. demonstrated that dinB is not a member of the canonical SOS regulon in *P. aeruginosa* and suggested that the lack of direct LexA-dependent regulation of this gene may be common among bacterial species. This observation remains controversial, as other studies have functionally linked dinB to the SOS response [25]. A plausible explanation is that *dinB* expression is activated through indirect mechanisms that operate under conditions of severe or prolonged DNA damage, rather than through direct LexA-mediated derepression. Studies indicate that an important role in this process is played by the stress factor RpoS (σS) [26]. These findings suggest that SOS regulation in *P. aeruginosa* involves multiple regulatory layers and is considerably more complex than the well-established LexA-centered SOS mechanism described in *E. coli*. Nevertheless, in addition to *dinB*, ciprofloxacin exposure led to the increased expression of three genes encoding inducible DNA polymerases: PA0923 (3.7-fold induction), PA0670 (*imuB*; 6.3-fold induction), and PA0669 (*dnaE2*; 1.9-fold induction). PA0923 encodes a Y-family DNA polymerase homologous to DinB, whereas *imuB* and *dnaE2* encode components of a polymerase complex implicated in error-prone, damage-tolerant DNA synthesis [11]. Taken together, these transcriptomic data indicate that ciprofloxacin exposure promotes a reprogramming of DNA metabolism, shifting the cell away from canonical high-fidelity replication toward a state that permits the engagement of inducible, error-tolerant polymerases.

A subset of the analyzed studies enabled a direct comparison of cellular responses in *P. aeruginosa* to β-lactam antibiotics targeting penicillin-binding proteins and to fluoroquinolones that induce DNA damage. These studies revealed pronounced qualitative and quantitative differences in SOS activation, downstream transcriptional programs, and phenotypic outcomes depending on the antibiotic class. Ceftazidime primarily inhibits PBP3, leading to defective septation, filamentation, and cell wall stress without directly damaging DNA. Importantly, the removal of ceftazidime resulted in the rapid resumption of growth, indicating that PBP3 inhibition induces a reversible adaptive state rather than irreversible cellular damage [24]. Despite these distinct primary targets, ceftazidime was shown to activate components of the SOS response even at subinhibitory concentrations, albeit through mechanistically distinct pathways compared with fluoroquinolones. Interestingly, in this particular study, ciprofloxacin did not induce *dinB* expression. However, this observation may be attributable to the relatively low antibiotic concentration used, which may have been insufficient to elicit full induction of the SOS regulon. In contrast, ceftazidime at subinhibitory concentrations leads to a more than 3-fold increase in *dinB* expression after five hours [24]. A common feature is a reduction in the expression of genes involved in global energy metabolism after exposure to both antibiotics, consistent with a broader metabolic slowdown associated with antibiotic stress. At the same time, several key differences in the global cellular response to these two antibiotics were observed. Most notably, ceftazidime attenuated the toxicity of ciprofloxacin by suppressing the expression of pyocin genes, thereby limiting a major SOS-associated cytotoxic output. Collectively, these findings underscore that the SOS response does not represent a uniform transcriptional program but rather a flexible signaling network that is integrated with other stress responses, such as cell wall damage. Accordingly, SOS activation triggered by PBP3 inhibition exhibits a fundamentally different character from that induced by fluoroquinolones. Another important distinction concerns the regulation of mobile genetic elements. Ciprofloxacin exposure led to a marked increase in the expression of prophage-related genes, transposases, and plasmid-associated functions, indicative of activation of mobile genetic elements. In contrast, ceftazidime elicited the opposite effect, repressing the expression of these genetic modules.

### 3.1. Role of RecA and LexA in SOS Activation and Antibiotic Susceptibility

Across all the analyzed studies, RecA emerged as an essential determinant of SOS response activation. RecA mutants consistently failed to induce SOS genes following their exposure to fluoroquinolones or other genotoxic agents, a finding confirmed at both the transcriptomic and protein levels [20,23]. The genetic complementation of *recA* fully restored SOS inducibility, thereby demonstrating a direct and causal role of RecA in both homologous recombination (HR) and initiating the SOS regulatory cascade. Beyond its regulatory function, RecA plays a critical role in survival under fluoroquinolone stress, as recA mutant strains show both larger inhibition zones and lower ciprofloxacin MIC values [27]. Specifically, the deletion of *recA* resulted in an approximately four-fold increase in susceptibility to ciprofloxacin and ofloxacin compared with the wild-type strain [20]. The restoration of a functional *recA* allele reverted the MIC values to those observed in the parental strain, further underscoring the contribution of RecA to antibiotic tolerance. Importantly, additional studies corroborated the pivotal role of RecA in ciprofloxacin tolerance and further implicated this protein in the emergence of resistant mutants, linking SOS activation not only to short-term survival but also to the evolutionary trajectories of antibiotic resistance [27]. It appears that *recA* plays a protective role during the early phases of antibiotic exposure. In contrast, the absence of functional RecA in bacterial populations in the stationary phase does not result in detectable differences in long-term survival. Nevertheless, recA-deficient cells exhibit pronounced morphological alterations, indicating that RecA influences cellular physiology beyond its role in survival per se [28]. These observations suggest that sustained activation of the SOS response is not a decisive determinant of long-term survival in *P. aeruginosa* under antibiotic stress. Instead, the susceptibility of stationary-phase cells is likely driven by metabolic activity and the associated generation of reactive oxygen species. Consistent with this interpretation, supplementation with antioxidants has been shown to enhance bacterial survival during exposure to levofloxacin, implicating oxidative stress as a major contributor to antibiotic-mediated lethality in non-growing populations [28].

The use of LexA variants defective in autocatalytic cleavage, such as LexA(S125A), enabled the functional separation of the SOS response from other cellular stress response pathways. These non-cleavable LexA mutants failed to induce SOS genes upon exposure to ciprofloxacin; however, their susceptibility to fluoroquinolones, as assessed by MICs, remained largely unchanged. This observation indicates that RecA plays a central role in determining cellular sensitivity to fluoroquinolones, whereas LexA, the second principal regulator of the SOS network, appears to be less critical in this context. Collectively, these findings demonstrate that canonical SOS activation is dispensable for baseline fluoroquinolone susceptibility and instead primarily contributes to stress adaptation, regulatory reprogramming, and longer-term cellular responses rather than directly mediating antibiotic lethality [11].The lack of an effect of functional LexA on direct susceptibility to ciprofloxacin is further supported by additional studies. The absence of functional LexA was associated with a reduction in short-term cellular fitness, as wild-type strains were shown to slightly outcompete isolates carrying mutations in the *lexA* gene [29]. These findings indicate that LexA contributes to the maintenance of cellular viability on a short-term timescale, while simultaneously underscoring the complexity and multiplicity of mechanisms that collectively determine bacterial survival in the presence of antibiotics. Taken together, these observations suggest that, although the SOS response influences fitness and stress adaptation, it does not directly dictate the immediate survival of individual *P. aeruginosa* cells during antibiotic exposure.

An additional level of SOS response regulation was described by Breidenstein et al., who demonstrated a critical role for the ATP-dependent Lon protease in modulating SOS activation, ciprofloxacin susceptibility, and mutagenesis in *P. aeruginosa*. Mutants lacking *Lon* exhibited a markedly reduced induction of the SOS regulon following exposure to ciprofloxacin. Notably, *RecA* expression was approximately 2.5-fold lower in the *Lon* mutant compared to the wild-type PAO1 strain [30]. SulA, a canonical SOS-regulated cell division inhibitor controlled by the LexA repressor, represents one of the putative molecular targets of Lon, although direct evidence for this interaction in *P. aeruginosa* remains limited. In addition, Lon has been proposed to indirectly influence RecA activity by modulating the stability of negative regulators such as RecX or components of the RecFOR pathway, thereby enabling full SOS activation. Consequently, the absence of Lon results in a dysregulated and functionally impaired SOS response. Taken together, these findings suggest that Lon fine-tunes the magnitude and duration of the SOS response.

### 3.2. Antibiotic-Induced Mutagenesis and Its Dependence on the SOS Response

Both in vitro and in vivo studies have demonstrated that exposure to fluoroquinolones results in a significant increase in mutation frequency, typically quantified by the emergence of mutants resistant to unrelated antibiotics such as rifampicin. This mutagenic effect is strongly dependent on RecA and an intact SOS response, as mutation rates are markedly reduced or completely abolished in recA-deficient strains or in strains carrying non-inducible lexA alleles [18,31]. In the case of *P. aeruginosa*, the data are more inconsistent. Hocquet et al. demonstrated that metronidazole, at concentrations corresponding to approximately 1/80 of the MIC, increased the frequency of *P. aeruginosa* mutants resistant to ciprofloxacin and amikacin through the induction of the SOS response [32]. Although metronidazole is not clinically effective against *P. aeruginosa*, its widespread use, most notably in the treatment of *Helicobacter pylori* associated gastritis, results in substantial environmental exposure, particularly in hospital settings. Although these observations raise important concerns, the clinical relevance of metronidazole-induced SOS activation likely depends on local drug concentrations, microbiome composition, and treatment duration, and has not yet been fully quantified in patient populations. Transcriptomic analyses revealed the increased expression of *recA* and *lexA* following metronidazole exposure. Concomitantly, a significant increase in the number of ciprofloxacin-resistant (2.8-fold) and amikacin-resistant (15.4-fold) isolates was observed. In contrast, recA deletion mutants did not exhibit a comparable increase in resistance to either antibiotic [32]. Sanders et al. indicate that relief of LexA-mediated repression does not directly translate into increased mutagenesis; instead, elevated expression of translesion synthesis (TLS) polymerases is the critical determinant of this effect [25]. However, bacteria harboring non-functional LexA variants exhibited a significantly reduced mutation frequency following antibiotic exposure, underscoring the contribution of LexA-dependent SOS regulation to antibiotic-induced mutagenesis [29].

The *dinB* gene encodes DNA polymerase IV (Pol IV), a member of the Y-family of DNA polymerases whose expression is induced in response to DNA damage. Owing to its ability to tolerate lesions in the DNA template, Pol IV plays an important role in maintaining replication continuity under genotoxic stress conditions. The overexpression of *dinB* resulted in an approximately 2.6-fold increase in the frequency of rifampicin-resistant mutants [25]. DinB is thought to participate in relatively accurate, low-mutagenic translesion DNA synthesis across DNA adducts. Nevertheless, dinB preferentially catalyzes erroneous transversions and frameshift mutations, particularly within homopolymeric sequences [25]. This phenomenon is clinically relevant, as similar mutations have been identified in the *mucA* gene among *P. aeruginosa* isolates obtained from cystic fibrosis patients [33]. Consequently, frameshift mutations may promote bacterial adaptation, increased virulence, and the transition of infection to a chronic state. The activity of dinB likely contributes to cell survival, as mutants lacking dinB exhibit an increased sensitivity to nitrofurazone and 4-nitroquinoline-1-oxide [25]. DinB appears to participate in relatively accurate, low-mutagenic TLS, and thus protects cells from death following exposure to genotoxic agents, without leading to a significant increase in mutation frequency. Nevertheless, Fahey et al. demonstrated that the number of ciprofloxacin-resistant mutants was significantly reduced in strains carrying a mutation in the *dinB* gene, whereas the restoration of a functional copy of this gene resulted in an increased mutation frequency. Mutations conferring resistance to ciprofloxacin appear to arise through a complex pathway involving both the activation of the SOS response and engagement of global stress response mechanisms. Moreover, dinB and another inducible translesion DNA polymerase, ImuC, seem to function in a coordinated manner. The decrease in mutagenesis observed in *dinB* deletion strains was not compensated by overexpression of the imuBC operon. Similarly, the reduced mutation frequency in *imuC* mutant strains was not restored by *dinB* overexpression. In contrast, in *imuB* deletion strains, the overexpression of *dinB* led only to a partial restoration of mutagenic activity [26].

At the same time, detailed mechanistic studies have demonstrated that fluoroquinolone-induced mutagenesis does not always require the involvement of classical error-prone DNA polymerases. In one pivotal study, deletion of *dinB*, *polB*, and *imuC*, either individually or in combination, did not abolish ciprofloxacin-induced mutagenesis. Furthermore, the mutation of *recA* also failed to affect the frequency of antibiotic-induced mutations [20]. Nonetheless, important methodological differences between the studies may have influenced the observed outcomes. In particular, the concentration of the antibiotic used was a critical factor. The subinhibitory concentration applied in the study by Mercolino et al. [20] may have been insufficient to generate resistant mutants, whereas higher concentrations, such as those used by Fahey et al. [26], revealed a significant dependence of mutagenesis on SOS system activation and the induction of TLS polymerases. The type of antibiotic used for mutagenesis induction is also of key importance. Metronidazole, whose active metabolites bind directly to DNA, markedly increased the frequency of resistant colonies [32]. In contrast, exposure to fluoroquinolones, which disrupt the replication machinery, may additionally activate alternative alarm response pathways [26]. Nevertheless, the study by Mercolino et al. provides evidence that, in *P. aeruginosa*, mutagenesis may proceed via an alternative mechanism independent of RecA and classical SOS induction. The involvement of additional mechanisms in the response to genotoxic stress is further supported by the findings of Fahey et al. A crucial role has been attributed to the general stress response sigma factor RpoS, as the number of mutants was significantly reduced in strains deficient in this regulator. Notably, this reduction was compensated by *dinB* overexpression, suggesting that the regulation of DinB is complex and depends on both SOS activation and the global stress response [26]. Other antibiotics, such as ceftazidime, have also been shown to contribute to SOS response activation and increased mutation frequency. A five-hour exposure to ceftazidime at a concentration corresponding to two-fold MIC resulted in nearly a five-fold increase in the number of resistant mutants [24]. Nevertheless, the profile of SOS activation varies depending on the nature of the stress-inducing agent. The available literature data challenge the direct extrapolation of the classical SOS response model established in *E. coli* to *P. aeruginosa* and instead point to the existence of alternative, species-specific mechanisms of genetic variability generation. Moreover, evolutionary studies indicate that SOS-induced mutagenesis is more likely a by-product than the primary adaptive function of this system. In this context, SOS activation does not appear to accelerate the evolution of antibiotic resistance, as long-term analyses spanning approximately 200 successive bacterial generations revealed no correlation between SOS-induced mutagenesis and the degree of adaptation to ciprofloxacin [29]. Studies also indicate the presence of key genetic differences that may underlie the distinct ciprofloxacin susceptibility profiles of clinical isolates compared with reference strains, as well as differences in mutation frequency. Clinical isolates were shown to exhibit a lower mutagenic potential despite ciprofloxacin exposure when compared with the reference strain PA14. Notably, the highest number of functional alterations was identified in the *imuB* and *recX* genes, both of which are regulated by RecA [21]. Moreover, the Lon protease appears to regulate antibiotic-induced mutagenesis. A strain carrying a mutation in the *lon* gene almost completely lost the ability to undergo ciprofloxacin-induced mutagenesis, whereas the complementation of the gene restored the observed phenotype [30].

### 3.3. Antibiotics as Indirect Modulators of Mutagenesis

A key conclusion drawn from the reviewed studies is that antibiotics lacking direct activity against *P. aeruginosa* may nevertheless indirectly induce the SOS response and promote the emergence of antibiotic resistance. Metronidazole, despite its lack of bactericidal activity against this species, was shown to induce *recA* and *lexA* expression, resulting in a significant increase in the frequency of mutants resistant to ciprofloxacin and amikacin. This effect was entirely dependent on RecA and was absent in *recA* deletion mutants [32]. Similarly, a clinical study provided evidence that antibiotic therapy administered in vivo can activate the SOS response and drive class 1 integron rearrangements, leading to the expression of previously silent resistance genes [34]. The removal of the integron cassette repressing blaOXA28 expression occurred exclusively under conditions of an active SOS response, with effects observed in vivo and confirmed by in vitro analyses. SOS-dependent integron activation resulted in expression of the gene encoding the OXA-28 β-lactamase, ultimately leading to the development of resistance to ceftazidime.

It was also demonstrated that amikacin applied at subinhibitory concentrations significantly attenuated SOS activation. Its administration resulted in more than a two-fold reduction in *recA* expression and almost complete suppression of ciprofloxacin-induced mutagenesis [27]. These findings indicate that combination therapy may substantially limit the evolutionary trajectory toward resistance. This conclusion is supported by studies examining the combined use of ciprofloxacin and meropenem, a β-lactam antibiotic, in the context of MDR strains. Concurrent exposure to both agents at concentrations corresponding to 0.5 × MIC led to decreased *lexA* expression. Importantly, from a clinical resistance perspective, this combination also reduced the expression of *ampC* and *oprD*, genes directly associated with antibiotic resistance [35]. Thus, combination therapy may weaken bacterial protective systems.

Clinically relevant observations were also obtained from studies employing *P. aeruginosa* isolates derived from cystic fibrosis patients. Prolonged incubation in the presence of subinhibitory antibiotic concentrations resulted in a significant reduction in susceptibility in approximately 22% of clinical isolates. This phenotypic shift was accompanied by genetic alterations, including mutations in genes encoding efflux pump regulators (*mexR*, *mexZ*, *mexS*, and *nalC*) as well as in *fusA1*, a gene associated with aminoglycoside resistance [36].

Collectively, these findings provide important insights into the determinants of antibiotic treatment efficacy and underscore the critical importance of achieving sufficiently high drug concentrations at the site of infection. Subinhibitory antibiotic exposure selects for a non-classical spectrum of resistance-associated mutations, and the selective mechanisms operating under these conditions differ fundamentally from those observed at higher antibiotic doses.

### 3.4. The SOS Response and Biofilm Formation, Tolerance, and Virulence

Several studies have demonstrated that the activation of the SOS response affects not only mutagenesis but also the phenotypic traits of bacterial populations, including biofilm formation and virulence. Subinhibitory concentrations of ciprofloxacin were shown to stimulate biofilm development in an LexA-dependent manner and were associated with increased cellular motility [37]. LexA functions as a negative regulator of motility; therefore, its autocatalytic cleavage, a hallmark event during SOS regulon activation, is essential for flagellar activity and surface colonization. In strains with an impaired SOS response, antibiotic-induced biofilm formation was not observed, and the synthesis of flagella-associated proteins was significantly reduced [37]. This study indicates that the SOS response acts as a key regulator of DNA damage–induced biofilm formation, and that maintenance of proper cellular motility, which is dependent on an intact SOS system, is critical during the early stages of biofilm development.

The activation of the SOS response also resulted in the increased production of outer membrane vesicles (OMVs). Importantly, OMV-producing bacteria exhibit significantly enhanced cytotoxicity toward host cells. This effect was partially LexA dependent, indicating a direct link between the genotoxic stress response and the modulation of bacterial pathogenicity [38]. The dependence of OMV production on SOS activation was further supported indirectly by studies examining the combined use of subinhibitory concentrations of ciprofloxacin and meropenem. This combination attenuated alarm response activation and concomitantly reduced OMV production [35].

Importantly, the relationship between SOS activation and virulence in *P. aeruginosa* appears to be antibiotic specific. A key divergence between β-lactams and fluoroquinolones has been observed in the regulation of pyocin synthesis. Pyocins are bacteriocins produced by *Pseudomonas* species that function as narrow-spectrum protein toxins targeting closely related bacterial strains. They are typically released in an SOS-dependent manner and often cause producer cell lysis, making their production a potent yet self-destructive competitive strategy. Through the selective killing of competing bacteria, pyocins contribute to niche dominance, population structuring, and virulence in polymicrobial environments. The exposure to fluoroquinolones results in the strong induction of pyocin operons, leading to increased cell lysis and enhanced bactericidal activity at the population level. In contrast, treatment with ceftazidime causes pronounced repression of pyocin genes despite the partial activation of the SOS response [24]. Functional assays demonstrated that such repression, related to PBP3 inhibition, reduced pyocin-mediated killing and antagonized ciprofloxacin-induced toxicity during combination therapy. This effect was dependent on intact pyocin loci and was absent in pyocin-deficient mutants, confirming that the antibiotic-specific modulation of SOS outputs directly influences cell death pathways [24]. In the context of *P. aeruginosa* virulence, the regulation of pyocin production represents a classical “double-edged sword” strategy—effective in eliminating competing bacteria but ultimately leading to lysis of the producer cell. Such lysis is accompanied by the release of additional toxins, metabolites, and extracellular DNA, which may exacerbate host inflammation and contribute to tissue damage. Pyocin production in *P. aeruginosa* is tightly controlled by the PrtR–PrtN regulatory system, which is functionally linked to the SOS response. Under genotoxic stress, the activation of RecA triggers proteolytic cleavage of the PrtR repressor, resulting in the derepression of the prtN gene. PrtN, acting as a transcriptional activator, initiates expression of pyocin biosynthesis genes, ultimately leading to lysis of the producer cell and release of the toxins.

However, exposure to ciprofloxacin reveals an additional, parallel layer of regulation. In addition to inducing the SOS response and promoting PrtR degradation, ciprofloxacin increases PrtR expression. Elevated PrtR levels enable the partial re-establishment of prtN repression even under conditions of active SOS signaling. As a result, a dynamic balance emerges between derepression and the secondary inhibition of the pyocin pathway. Consequently, the bacterial population retains the ability to eliminate competitors through localized pyocin production while minimizing the biological cost associated with loss of toxin-producing cells. The analysis of clinical strains revealed increased pyocin production compared with the reference strain PA14, which may partially account for the greater susceptibility of these isolates to ciprofloxacin [21]. Moreover, the ATP-dependent protease Lon has emerged as another crucial regulator of pyocin synthesis. Strains lacking the *lon* gene exhibit deregulation of the RecA/PrtR/PrtN regulatory axis, which results in a marked reduction in pyocin production [30]. Importantly, Lon is not required for SOS induction per se, but rather for the proper execution and coordination of SOS-dependent pathways. Although the downregulation of RecA-controlled pyocin genes in a lon mutant would theoretically be expected to decrease susceptibility to ciprofloxacin, the observed hypersensitivity of these strains indicates that Lon is essential for effective SOS-mediated stress adaptation beyond pyocin regulation alone.

Concurrently, PrtR plays a second yet equally important role in virulence regulation, as it is required for expression of the type III secretion system (T3SS). PrtR acts indirectly by repressing *ptrB*, a gene encoding a T3SS inhibitor. Consequently, the degradation of PrtR under conditions of strong SOS activation also leads to the deregulation of T3SS, a critical effector of acute virulence and bacterial-mediated neutrophil killing. The functional consequence of this autoregulatory circuit is the maintenance of a balance between virulence and cell survival. Stable PrtR levels allow for sustained T3SS activity within the host environment, facilitating colonization and immune cell elimination, while simultaneously limiting excessive pyocin production and associated cell lysis. Under ciprofloxacin exposure, this mechanism additionally promotes increased bacterial tolerance, particularly within biofilms, by suppressing the suicidal pyocin response [39]. In this framework, PrtR emerges not as a simple component of the SOS cascade but as a central regulator of stress homeostasis, integrating genotoxic signals, host immune responses, and virulence regulation. This mechanism enables *P. aeruginosa* to maintain pathogenic potential under conditions of oxidative stress and fluoroquinolone exposure while minimizing the biological costs associated with uncontrolled SOS activation and excessive bacteriocin production. Moreover, PrtR enhances biofilms’ tolerance to ciprofloxacin, as both prtR overexpression and prtN deletion significantly increased cell survival within biofilms. These data demonstrate that ciprofloxacin-induced biofilm loss is not a direct consequence of antibiotic exposure but rather results from pyocin-dependent cell lysis, which can be attenuated by the PrtR-mediated, SOS-dependent repression of the pyocin pathway [39]. Studies have demonstrated that the SOS response is not overexpressed in biofilms. Instead, the increased tolerance of biofilms to ciprofloxacin appears to be driven by enhanced expression of the hypoxia-associated anr regulon, genes involved in osmotic stress responses, and components of quorum-sensing pathways. In addition, bacteria growing in the biofilm state exhibit significantly elevated expression of the global stress response regulator rpoS [40]. These findings indicate that antibiotic tolerance of *P. aeruginosa* biofilms, particularly to ciprofloxacin, results from overlapping stress responses associated with hypoxia and starvation rather than from classical resistance mechanisms.

### 3.5. The SOS Response in the Context of the Stationary Phase and Persister Cells

Recent studies have demonstrated that during the stationary phase, *P. aeruginosa* retains substantial metabolic and transcriptional activity, paradoxically rendering it susceptible to fluoroquinolone treatment. Under these conditions, RecA activity and SOS induction did not determine cell survival. Instead, bacterial death occurred predominantly during antibiotic exposure rather than after drug removal, representing a significant divergence from the classical *E. coli* model [28]. Indeed, in *E. coli*, the SOS response plays an important role in the formation of persister cells [19]. Single-cell analyses of *P. aeruginosa* persisters have revealed that most of these cells avoid the formation of DNA double-strand breaks (DSBs) rather than relying on their repair [41]. Only a small fraction of persister cells exhibited signs of DSBs, and their presence delayed, but did not prevent, the resumption of growth following antibiotic withdrawal. Importantly, persister cells did not display increased resistance to levofloxacin, indicating that SOS activation is not a key determinant of population recovery after antibiotic-induced stress [].

### 3.6. Antibiotic- and Stress-Induced Modulation of the SOS Response

Although fluoroquinolones represent the most extensively studied inducers of the SOS response in *P. aeruginosa*, the studies summarized in Table 1 demonstrate that SOS signaling can be influenced by a broader range of antibiotics and environmental stressors. These include agents with distinct primary targets, such as β-lactams (e.g., ceftazidime), aminoglycosides (e.g., amikacin), carbapenems (e.g., meropenem), and even antibiotics that do not exhibit direct antibacterial activity against *P. aeruginosa*, such as metronidazole. Despite their different mechanisms of action, these agents may modulate the SOS network through indirect stress pathways, including oxidative stress, metabolic perturbation, or disruption of DNA replication processes [24,27,32,35].

Importantly, the studies collected in Table 1 indicate that the interaction between antibiotic exposure and SOS signaling is not uniform but may produce several distinct outcomes. In some cases, antibiotic exposure directly induces SOS activation and increases mutation frequency, as observed for fluoroquinolones or metronidazole [20,32]. In other situations, antibiotics primarily modulate the magnitude or consequences of SOS activation without necessarily increasing mutagenesis. For example, combination therapies involving aminoglycosides or carbapenems may suppress SOS-associated mutagenesis or alter the transcription of key regulatory genes such as *recA* or *lexA* [27,35]. These findings highlight that antibiotic interactions with the SOS network may either promote or attenuate stress-induced genetic variability.

The data summarized in Table 1 also illustrate that SOS signaling in *P. aeruginosa* is closely integrated with other stress response pathways and physiological processes. The activation of the SOS system may influence diverse phenotypes, including virulence regulation, biofilm formation, membrane vesiculation, and stress adaptation mechanisms mediated by regulators such as RpoS or Lon [26,30,37,38]. In addition, SOS activation may occur in response to non-antibiotic stressors, such as ultraviolet radiation or chemical DNA-damaging agents, further emphasizing the role of this regulatory network as a general genomic stress response.

Taken together, the available evidence demonstrates that the SOS response in *P. aeruginosa* should not be viewed solely as a reaction to fluoroquinolone-induced DNA damage. Instead, it represents a broader and highly context-dependent regulatory system that integrates signals from diverse antibiotic classes and environmental stressors, ultimately shaping bacterial adaptation, mutagenesis, and phenotypic plasticity.

**Table 1:** Antibiotic- and stress-induced SOS responses in *Pseudomonas aeruginosa*.

Antibiotic/Stress	Experimental Model	SOS Activation	Mutagenesis	Key Mechanistic Insight	Ref.
NOR/UV	PAO (*RecA*^+^/*RecA*^−^)	Yes	ND	RecA autoregulation; first evidence of inducible SOS stress response	[23]
CIP (8× MIC)	PAO1, *lexA*(S125A)	Yes	ND	Narrow LexA regulon (~15 genes); LexA does not determine MIC but contributes to adaptive potential	[11]
CIP (sub-MIC)	PAO1 vs. *lexA*(S125A)	Yes	ND	SOS increases fitness but does not accelerate resistance evolution	[29]
CIP	PAO1 Δ*dinB* Δ*imuBC* Δ*rpoS*	Yes	Yes	Cooperation between SOS and RpoS; polygenic mutagenesis	[26]
FQs	PAO1 Δ*recA* Δ*TLS*	Yes	Yes	SOS-independent mutagenesis challenges canonical *E. coli* model	[20]
UV, NFZ, 4-NQO	PAO1 Δ*dinB*	Yes	Yes	DinB mediates frameshifts and transversions; dual protective and mutagenic role	[25]
MET (1/80 MIC)	PA14 Δ*recA*	Yes	Yes	RecA-dependent mutagenesis; indirect induction of resistance to CIP and AMI	[32]
CIP (sub-MIC)	PA14 vs. CF isolates	Yes	Yes	Clinical isolates show distinct mutation patterns (imuB, recX, and pyocin genes)	[21]
MET/CAZ	MDR clinical isolates	Yes	Yes	Integron activation (intI1); clinically relevant resistance emergence	[34]
CIP + AMI	PAO1, *RecA* mutant	Yes	Yes	AMI suppresses recA expression and reduces mutagenesis	[27]
CIP + MER (sub-MIC)	MDR isolates	Yes	ND	Repression of lexA expression and reduced OMV production	[35]
CIP	PAK Δ*prtR*, Δ*prtN*, Δ*prtR*	Yes	ND	PrtR-dependent regulation balances virulence and survival	[39]
CIP/TOB	PAO1	Yes (not essential)	ND	Tolerance linked to hypoxia stress, RpoS and stationary phase physiology	[40]
sub-MIC CIP	PAO1 vs. *Lex*AN	Yes	ND	LexA regulates motility; promotes biofilm initiation	[37]
CIP	PAO1 vs. *Lex*AN	Yes	ND	Increased OMV production; SOS modulates virulence	[38]
CIP	PAO1 Δ*lon*	Yes	Yes	Lon protease regulates SOS activation and mutagenesis	[30]
LVX	PAO1 Δ*recA*	ND	ND	Survival determined by oxidative stress rather than SOS activation	[28]
LVX	PAO1, PA14	No	ND	Persister survival depends on avoidance of DNA double-strand breaks	[41]
CIP vs. CAZ	PAO1	Yes	ND	β-lactams modulate fluoroquinolone toxicity via stress-dependent SOS branches	[24]
CIP, TOB, CAZ, MER (sub-MIC)	PAO1, PA14, CF isolates	ND	Yes	Sub-MIC exposure promotes resistance-associated mutations	[36]

Abbreviations: NOR: norfloxacin; CIP: ciprofloxacin; MET: metronidazole; AMI: amikacin; MER: meropenem; TOB: tobramycin; CAZ: ceftazidime; LVX: levofloxacin; FQs: fluoroquinolones; NFZ: nitrofurazone; 4-NQO: 4-nitroquinoline-1-oxide; TLS: translesion synthesis; MIC: minimal inhibitory concentration; ND: not determined; CF: cystic fibrosis; LexAN: non-cleavable; and Δ: deletion.

## 4. Clinical and Translational Implications

*Pseudomonas aeruginosa*, a Gram-negative, non-fermenting rod, is one of the most common etiological agents of diabetic foot infections. Such infections significantly impair wound and ulcer healing, thereby worsening patients’ prognosis and markedly increasing the risk of lower limb amputation. The rising prevalence of antibiotic resistance and the increasing detection of multidrug-resistant strains underscore the urgent need for novel and effective therapeutic strategies targeting this pathogen. In patients with diabetes, timely and accurate diagnosis, as well as effective treatment, are often considerably hindered by coexisting complications, including peripheral neuropathy and peripheral vascular disease [42]. Consequently, healthcare systems face an urgent need to implement targeted and adequate interventions for this particularly vulnerable patient population. The SOS response in *P. aeruginosa* is an adaptive system rather than a classical survival mechanism. Accumulated and critically analyzed data clearly indicate that the SOS response in *P. aeruginosa* serves a fundamentally different role than that classically attributed to this system based on the *E. coli* model. In contrast to enterobacteria, where SOS directly promotes cell survival following DNA damage, in *P. aeruginosa,* this response is not a major determinant of either tolerance or susceptibility to fluoroquinolones. This conclusion is supported by multiple studies employing non-inducible *lexA* mutants, which, despite a blocked SOS response, exhibit MIC values comparable to those of wild-type strains and similar killing kinetics against ciprofloxacin and levofloxacin [11,29]. Moreover, the absence of a functional SOS response does not result in a significant reduction in the long-term adaptive capacity, as assessed by mutation frequency and the emergence of resistant variants [29]. These findings suggest that, in *P. aeruginosa*, the SOS response primarily enhances short-term population robustness and adaptive potential rather than serving as a direct evolutionary strategy. This distinction between survival and evolvability represents one of the central conclusions of the present review. One of the most striking features of the SOS response in *P. aeruginosa* is its exceptionally narrow regulatory scope. In contrast to the extensive SOS regulon of *E. coli*, which encompasses dozens of genes involved in diverse DNA repair pathways, the LexA regulon in *P. aeruginosa* comprises only approximately 15 genes [11]. These genes are primarily associated with homologous recombination (e.g., *recA*, *recN*, and *recX*) and inducible DNA polymerases (*imuB* and *dnaE2*), while many canonical SOS genes, such as components of nucleotide excision repair, are notably absent from LexA regulation. This regulatory architecture has important biological implications. First, these observations indicate that in *P. aeruginosa*, adaptive responses to stress are mediated not only by the canonical SOS pathway but also by additional stress response networks. In particular, the global stress response governed by the sigma factor RpoS plays a critical role by integrating environmental cues associated with growth arrest, nutrient limitation, and oxidative stress, thereby promoting bacterial survival under antibiotic pressure [26]. In this respect, *P. aeruginosa* represents an alternative SOS model that should not be interpreted through the paradigm established for *E. coli*.

The reviewed studies consistently demonstrate that sublethal concentrations of fluoroquinolones increase the mutation frequency in *P. aeruginosa*. This phenomenon is largely dependent on RecA and SOS activation; however, the underlying molecular mechanisms differ substantially from classical SOS-dependent mutagenesis. Key studies have shown that deletion of genes encoding error-prone DNA polymerases, such as *dinB*, *polB*, or *imuC*, does not abolish fluoroquinolone-induced mutagenesis [20]. This indicates that the observed increase in genetic variability is not merely a consequence of derepressed translesion synthesis polymerases, but rather arises from complex interactions among DNA replication, recombination, and DNA metabolism. Conversely, detailed biochemical and genetic analyses have revealed that DNA polymerase IV (DinB) in *P. aeruginosa* possesses genuine mutagenic potential, particularly in generating frameshift mutations within homopolymeric sequences [25]. Such mutations are especially relevant in the context of chronic adaptation, as they underlie alterations in regulatory genes (e.g., mucA) frequently observed in clinical isolates. A critical unresolved issue, however, is the LexA dependence of dinB expression, which has not been consistently demonstrated across all studies [11,25]. This variability suggests that the extent of SOS activation may depend on the nature and intensity of the stressor acting on the cell.

One of the most important and concerning conclusions emerging from the reviewed literature is that antibiotics can actively initiate processes leading to resistance development, even when they exhibit no direct antibacterial activity against *P. aeruginosa*. However, it should be emphasized that most experimental evidence discussed herein is derived from laboratory strains and controlled in vitro conditions, which may not fully capture the complexity of in vivo infections. Studies on metronidazole provide compelling evidence that this drug, through the induction of oxidative stress and secondary DNA damage, activates the SOS response, resulting in increased frequencies of mutants resistant to fluoroquinolones and aminoglycosides [32]. Of particular significance is a clinical study providing direct in vivo evidence that antibiotic therapy can trigger the SOS response within the patient, leading to the restructuring of class 1 integrons [34]. The unmasking of a “silent” resistance gene via integron cassette excision represents a landmark demonstration that the SOS response can additionally function as a phenotypic resistance switch rather than merely a generator of de novo mutations. This finding fundamentally alters the perception of risks associated with therapy and the use of antibiotics formally considered inactive against a given pathogen. Moreover, accumulating evidence indicates that the mutational profiles arising under subinhibitory antibiotic concentrations differ from those induced by antibiotics administered at growth-inhibitory doses [36].

Data concerning biofilms supports the notion that the SOS response is not a primary mechanism underlying biofilm tolerance to antibiotics, particularly ciprofloxacin. Instead, such tolerance arises predominantly from overlapping stress responses associated with hypoxia, growth arrest, and metabolic reprogramming, regulated by Anr, RpoS, and the stringent response [40]. Nevertheless, the SOS response may modulate specific stages of biofilms’ development, especially initiation, through the derepression of flagellum-dependent motility [37]. An intriguing extension of the classical SOS model is provided by studies on the PrtR regulator, which demonstrate that genotoxic stress responses are tightly interconnected with virulence regulation and energetically costly “suicidal” responses, such as pyocin production. PrtR functions as a stress homeostasis element, stabilizing the SOS response and preventing the excessive activation of pathways leading to cell lysis [39]. This mechanism underscores that the SOS response in *P. aeruginosa* is embedded within a broader regulatory network that encompasses virulence, biofilm formation, and host interactions. The SOS system also fulfills protective functions, and its complete inhibition may lead to unpredictable adverse effects, such as increased genomic instability or the selection of alternative adaptive pathways [30]. Furthermore, persister cells do not rely on SOS activation but rather on avoiding genetic damage altogether.

## 5. Challenges and Future Directions

Despite substantial progress in characterizing the SOS network in *P. aeruginosa*, several conceptual and translational challenges remain unresolved. First, most available data derive from laboratory reference strains and short-term in vitro models. As highlighted, clinical isolates frequently display distinct mutational spectra, altered RecA–ImuB regulation, and different prophage or pyocin profiles compared with PAO1/PA14 backgrounds. Systematic, strain-diverse and in vivo-relevant analyses are therefore essential to avoid the overgeneralization of laboratory findings. Second, the precise contribution of SOS activation to antibiotic-induced mutagenesis remains context dependent. Conflicting observations—ranging from clear RecA dependence to apparent SOS-independent mutagenesis—suggest that parallel stress pathways (e.g., RpoS-mediated responses, redox imbalance, and metabolic state) intersect with DNA damage signaling. Dissecting these regulatory layers will require further analyses, the use of real-time reporters, and the integration of transcriptomic, proteomic, and metabolomic datasets under clinically relevant antibiotic exposure conditions. Third, the therapeutic targeting of the SOS system presents both promise and uncertainty. Although the inhibition of RecA or LexA autocleavage in some species has been proposed to limit mutagenesis, evolutionary experiments indicate that blocking SOS does not universally prevent long-term adaptation in *P. aeruginosa*. Moreover, the SOS response modulates virulence traits, biofilm initiation, integron rearrangements, and outer membrane vesiculation; indiscriminate suppression may therefore produce unintended ecological or pathogenic consequences. Future work should evaluate SOS-targeted strategies within combination therapies, focusing not only on bactericidal activity but also on evolutionary trajectories, biofilm tolerance, and horizontal gene transfer. Finally, the relationship between antibiotic pharmacokinetics and subinhibitory exposure requires deeper investigation. Increasing evidence indicates that non-lethal drug concentrations—whether environmental or tissue specific—reshape stress signaling and resistance emergence. Integrating molecular SOS dynamics with pharmacodynamic modeling and infection site measurements represents a critical next step. Collectively, advancing the field will require moving beyond a binary “SOS on/off” framework toward a systems-level understanding of stress integration in *P. aeruginosa*. Such an approach may enable the rational design of therapies that suppress adaptive potential without destabilizing essential stress homeostasis.

## 6. General Conclusions

Antibiotics, particularly fluoroquinolones, but also agents formally considered inactive against *P. aeruginosa*, can act as potent evolutionary signals, triggering processes that paradoxically undermine therapeutic efficacy. A comprehensive understanding of the complex role of the SOS response is essential for the development of innovative therapeutic strategies that address not only pathogen eradication but also the control of bacterial adaptive potential. Collectively, the available evidence supports a model in which the SOS response in *P. aeruginosa* functions primarily as a short-term stress adaptation system, modulating susceptibility, virulence, and genetic plasticity in a context-dependent manner, while its contribution to long-term resistance evolution remains secondary and highly conditional.

## Data Availability

No new data were created or analyzed in this study. Data sharing is not applicable to this article.
